# Diabetic Retinopathy: Soluble and Imaging Ocular Biomarkers

**DOI:** 10.3390/jcm12030912

**Published:** 2023-01-24

**Authors:** Mariantonia Ferrara, Alessandra Loda, Giulia Coco, Piergiacomo Grassi, Silvia Cestaro, Sara Rezzola, Vito Romano, Francesco Semeraro

**Affiliations:** 1 Manchester Royal Eye Hospital, Oxford Road, Manchester M13 9WL, UK; 2 Department of Molecular and Translational Medicine, University of Brescia, Viale Europa 11, 25123 Brescia, Italy; 3 Department of Clinical Science and Translational Medicine, University of Rome Tor Vergata, 00133 Rome, Italy; 4 Northern Care Alliance NHS Foundation Trust, Whitehall St, Rochdale OL12 0NB, UK; 5 School of Medical Sciences, Faculty of Biology, Medicine and Health, The University of Manchester, Oxford Road, Manchester M13 9PL, UK; 6 Eye Clinic, Department of Medical and Surgical Specialties, Radiological Sciences, and Public Health, University of Brescia, 25121 Brescia, Italy; 7 ASST Civil Hospital of Brescia, 25123 Brescia, Italy

**Keywords:** corneal endothelial cell count, confocal microscopy, diabetic retinopathy, fluorescein angiography, ocular biomarkers diabetic retinopathy, optical coherence tomography, optical coherence tomography angiography, serum biomarkers diabetic retinopathy, ultra-widefield fundus photography

## Abstract

Diabetic retinopathy (DR), the most common microvascular complication of diabetes mellitus, represents the leading cause of acquired blindness in the working-age population. Due to the potential absence of symptoms in the early stages of the disease, the identification of clinical biomarkers can have a crucial role in the early diagnosis of DR as well as for the detection of prognostic factors. In particular, imaging techniques are fundamental tools for screening, diagnosis, classification, monitoring, treatment planning and prognostic assessment in DR. In this context, the identification of ocular and systemic biomarkers is crucial to facilitate the risk stratification of diabetic patients; moreover, reliable biomarkers could provide prognostic information on disease progression as well as assist in predicting a patient’s response to therapy. In this context, this review aimed to provide an updated and comprehensive overview of the soluble and anatomical biomarkers associated with DR.

## 1. Introduction

Diabetic retinopathy (DR) is the most common microvascular complication of diabetes mellitus (DM), and it represents the leading cause of acquired blindness in the working-age population in developed countries [1]. The disease is characterized by an initial, non-proliferative stage (NPDR) that manifests with increased vascular permeability due to damage to the retinal microvasculature, and, consequently, vascular leakage, lipidic exudates, areas of ischemia, and microaneurysms [2,3]. NPDR can progress into proliferative DR (PDR), which is characterized by a marked neovascularization and by the formation of fragile new blood vessels through the retina and into the vitreous humor. If untreated, DR can lead to vitreous hemorrhage, diabetic macular edema (DME), tractional detachment of the retina, and, eventually, blindness [2,4]. Patients with DR can be asymptomatic until advanced stages of the disease; thus, regular eye screenings play a crucial role in order to timely identify pathologic signs. Imaging techniques are fundamental tools in ophthalmology and their role for screening, diagnosis, classification, monitoring, treatment planning and prognostic assessment in several common ophthalmic diseases, including DR, is constantly expanding [5,6,7,8,9,10,11,12]. Importantly, the high resolution and sensitivity of these techniques can lead to detect microstructural subclinical changes, potentially improving the effectiveness of population-screening programs and facilitating an early diagnosis. Furthermore, newly identified biomarkers could also provide new insights into the pathogenesis of DR.

In this context, the identification of ocular and systemic biomarkers is crucial to facilitate the early diagnosis and to guide the risk stratification of diabetic patients; moreover, reliable biomarkers could provide prognostic information on disease progression as well as assist in predicting a patient’s response to therapy. This review aimed to provide a comprehensive overview of the soluble and anatomical biomarkers associated with diabetic retinopathy.

## 2. Methods

We performed a comprehensive literature review regarding ocular and serum biomarkers of diabetic retinopathy using PubMed, Cochrane and the Embase database up to October 2022, with no limit associated with the year of publication. The keywords used for this search were: corneal endothelial cell count; confocal microscopy; diabetic retinopathy; fluorescein angiography; ocular biomarkers diabetic retinopathy; optical coherence tomography; optical coherence tomography angiography; serum biomarkers diabetic retinopathy; ultra-widefield fundus photography. We included clinical studies with both prospective or retrospective design, whereas editorials, case reports, observations, expert opinions, letters to the editor and non-inherent studies were excluded.

## 3. Brief Overview on the Pathogenesis of Diabetic Retinopathy

Diabetic retinopathy is a multifactorial disease and several factors contribute to its onset, including hyperglycemia, hypoxia, inflammation, and oxidative stress. For a long time, DR has been considered as a vascular disorder due to the extensive involvement of vascular alterations in the pathogenesis of the disease; however, several studies have demonstrated that endothelial disfunction and microangiopathy are only one aspect of a more widespread retinal disfunction, affecting also glial and neuronal cells [13,14,15]. Indeed, long-term hyperglycemia activates numerous metabolic pathways involved in the production of reactive oxygen species (ROS) and pro-inflammatory mediators, which are in turn associated to leukostasis, disruption of cell–cell junctions, loss of endothelial cells and pericytes, and breakdown of the blood–retinal barrier BRB, resulting in vascular dysfunction, neurodegeneration and microglia activation [16]. In addition, hyperglycemia promotes the dysfunction and loss of the endothelial glycocalyx contributing to the increase in vascular permeability, capillary occlusion and leukostasis, and, thus potentially to atherothrombotic processes and DR progression [17,18]. The concomitant complement hyperactivation and the accumulation of immune cells and pro-inflammatory molecules into the retina due to the BRB breakdown contribute to DR progression further promoting retinal neurovascular damage and local chronic low-grade inflammation [19]. As the severity of the disease progresses, capillary non-perfusion leads to retinal ischemia, increasingly affecting larger areas of the retina [20]; as a consequence, the balance between pro-angiogenic and anti-angiogenic mediators is shifted, resulting in neovascularization [4,21].

## 4. Angiogenic and Inflammatory Mediators in Diabetic Retinopathy

**Figure 1:**
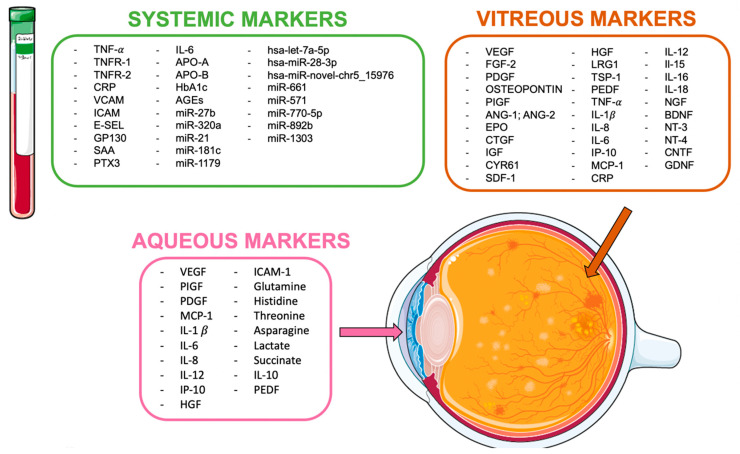
Soluble biomarkers in diabetic retinopathy.

Several studies investigated the presence of exploitable biomarkers by analyzing different biological fluids obtained from patients, such as vitreous humor, aqueous humor, and blood (Figure 1).

### 4.1. Vitreous Humor Biomarkers

Due to its proximity to the retina, the vitreous is deeply affected by the pathological events that occur during DR progression, and it undergoes structural and molecular alterations, which are reflected by a marked change of its proteomic profile [22]. A recent extensive analysis on 138 vitreous samples from eyes with DR identified over 1350 distinct proteins, with 230 proteins being more abundant in patients with PDR compared to NPDR, including angiogenic factors and inflammatory mediators, complement and coagulation cascade proteins, protease inhibitors, apolipoproteins, immunoglobulins, proteins involved in ROS production, and cell adhesion molecules [23].

In the diabetic retina, the balance between pro-angiogenic and anti-angiogenic mediators is shifted toward the establishment of a more pro-angiogenic microenvironment. Consistently, in diabetic vitreous, several pro-angiogenic mediators are upregulated, whereas some anti-angiogenic mediators are downregulated (Table 1) [24,25,26,27,28,29,30,31,32,33,34,35,36,37,38,39]. Consistently with the extensive role of inflammation in DR, several pro-inflammatory cytokines and chemokines are commonly found to be upregulated in the vitreous of patients with PDR (Table 1) [2,40,41,42,43]. Interestingly, increased levels of interleukin (IL) 1b, IL-18, and IL-6, as well as vascular endothelial growth factor (VEGF), have been shown to correlate with disease severity [43,44]. It is worth mentioning that patients with NPDR had significantly higher vitreous concentrations of neurotrophins when compared to patients with PDR, supposedly due to an increased production of neurotrophins by retinal glial cells, as an attempt to rescue neuronal cells during the early stages of DR [45,46]. Finally, the vitreous obtained from patients with PDR exerts a significant biological activity in several in vitro and in vivo experimental models, and it reflects the biological variability that occurs in patients as a consequence of different clinical parameters [47,48,49]; thus, the diabetic vitreous might be employed to guide drug discovery as well as to facilitate the selection of more personalized pharmacological treatments better suited to the clinical features of each patient.

### 4.2. Aqueous Humor Biomarkers

Aqueous humor might represent a useful tool to better characterize the ocular angio-inflammatory profile of diabetic patients and to monitor their response to therapy as proteins released from the diabetic retina diffuse from the vitreous humor into the aqueous [50]. In addition, the analysis of the aqueous is favored by the relative ease and safety of sample withdraw compared to vitreous [51].

Despite its high turnover rate, several pro-inflammatory and pro-angiogenic mediators have been found upregulated in the aqueous humor of patients affected by DR (Table 1) [52,53,54,55,56,57,58,59]. On the other hand, significantly lower levels of the anti-inflammatory cytokine IL-10 and of the anti-angiogenic factor pigment epothlium-derived factor (PEDF) have been associated with increased severity of DR and higher risk of developing DME [53,54,55,56,57,58,59,60,61,62]. In this context, the presence of DR seems also to be associated to higher levels of the long pentraxin 3, supporting the role of this protein in the local inflammatory reaction to hyperglycemia [60].

The metabolomic profile of aqueous humor revealed increased levels of the glucogenic amino acids glutamine, histidine, threonine, and asparagine in patients with DR compared to diabetic patients without DR [61]. This was paralleled by reduced levels of lactate and succinate, which was probably due to the mitochondrial damage that occurs in the diabetic retina [61].

The relative concentration of different aqueous biomarkers has been exploited to investigate and/or to compare the therapeutic effect of different drugs through measurement before and after a certain treatment. For instance, it has been reported that intravitreal triamcinolone acetonide resulted in a significative reduction in several angio-inflammatory mediators (i.e., IL-6, IP-10, MCP-1, PDGF-AA, and VEGF), whereas intravitreal bevacizumab led to reduced levels only of VEGF [54]. In addition, a progressive decrease in VEGF, placental growth factor (PlGF), and tumor necrosis factor (TNF) α levels has been demonstrated following panretinal photocoagulation (PRP), supporting the effectiveness of targeting hypoxic retinal areas for reducing the angio-inflammatory microenvironment of DR [54].

### 4.3. Serum and Plasma Biomarkers

A variety of mediators has been associated to the onset of microvascular complications in diabetic patients, including high concentrations of soluble VCAM, ICAM, E-selectin, glycoprotein 130, serum amyloid A, pentraxin 3, and IL6 [63,64,65,66]. In addition, high levels of TNF-α, as well as of soluble TNF receptors 1 and 2 (TNFR-1 and TNFR-2, respectively), have been found in serum of patients with DR, and they have been associated with disease progression and with an increased risk of developing PDR and DME; interestingly, while soluble receptors are usually regarded as TNF-antagonists, in this context, they represent a reservoir of circulating TNF-α [65,67]. Furthermore, high serum levels of CRP have been identified as predictive of developing retinal hard exudates and DME [68]. Moreover, an in-depth analysis of serum metabolic markers suggested that increased levels of apolipoprotein B (APO-B) and decreased levels of apolipoprotein A (APO-A) correlate to DR severity, whereas a high APO-B/APO-A ratio is positively associated to increased risk of developing DME [69]. Conversely, even though serum levels of VEGF are increased in patients with DR, they are not predictive of eye disease progression; however, the evidence that circulating levels of VEGF are significantly reduced in DR patients after intravitreal administration of the anti-VEGF aflibercept suggests a potential utility of dosing VEGF in plasma to monitor response to therapy [65].

Despite the identification of the above-mentioned biological mediators, the only validated biomarker for the prediction of DR onset and progression is glycated hemoglobin (HbA1c), confirming that a good glycemic control is effective in reducing the risk of DR and its complications [70]. Accordingly, higher baseline levels of advanced glycation-end products (AGEs) are significantly associated to increased risk of disease progression [71].

Recently, noncoding RNAs have emerged as a promising biomarker for the early diagnosis and monitoring of various diseases [72]. MicroRNAs (miRNAs) are highly conserved 19–25 nucleotide noncoding RNAs that regulate gene expression by blocking the translation of messenger RNAs [73]. When released into the circulation, miRNAs are very stable and they have a long lifespan, which makes them suitable for investigation [74]. miRNAs have been also implicated in the microvascular complications of diabetes, promoting inflammation and endothelial dysfunction [75]. Circulating miR-27b and miR-320a have been associated with increased risk of DR, probably exerting a pro-angiogenic function [75]. Moreover, increased levels of miR-21, miR-181c, and miR-1179 have been found in patients with PDR compared to NPDR; supposedly, they provide a distinct fingerprint for PDR with a moderate efficacy in discriminating between NPDR and more advanced disease [73]. Additionally, a panel of three circulating miRNAs, which includes hsa-let-7a-5p, hsa-miR-28-3p, and hsa-miR-novel-chr5_15976, has been reported as successful in discriminating between diabetic patients with or without DR as well as in distinguishing between early and severe DR [76]. Finally, high levels of miR-661, miR-571, miR-770-5p, miR-892b, and miR-1303 in diabetic patients have been associated with increased risk of microvascular complications [77].

## 5. Corneal Biomarkers

Anterior segment sequelae of DM are not as well defined as DR. However, up to 2/3 of patients can develop diabetic keratopathy [78,79]. Despite its limited and occasionally controversial nature, the available evidence suggests that corneal structural and biomechanical changes in diabetic eyes may be potential biomarkers in the early diagnosis of DM and its complications [80].

In general, all corneal layers may be affected by morphological and functional changes in diabetic eyes, and a variety of alterations have been described [81]. Indeed, the metabolic stress induced by chronic hyperglycemia activates several pathological pathways, resulting in endothelium damage, corneal edema, endothelial cells loss, progressive deprivation in corneal nerve fiber mass with consequent increased epithelial fragility, reduced epithelial cell density and corneal susceptibility to persistent epithelial defects, recurrent corneal ulcerations and infections [82,83,84]. In addition, due to the inability of the corneal endothelium to regenerate in response to the endothelial cell loss, compensatory morphological changes of the endothelial cells can be observed, such as increased cellular pleomorphism, polymegathism and decrease in the percentage of hexagonal cells (Hex) [83].

In this light, corneal parameters related to endothelial dysfunction and corneal neuropathy have been investigated as surrogate markers for DM. This section will focus on the main DM-associated corneal changes and, in particular, on the findings that each imaging technique allows us to analyze.

### 5.1. Corneal Thickness

Diabetes-related alterations in central corneal thickness (CCT) may be due to the endothelial damage and the subsequent unbalanced corneal hydration and corneal edema [83,85]. The studies analyzing CCT in diabetic and non-diabetic eyes are resumed in Table 2.

#### 5.1.1. Anterior Segment OCT

Studies with AS-OCT described an increased CCT in diabetic patients compared to controls (Table 2) [86,87,88]. It is worth noting that CCT measurements using AS-OCT may be higher than those obtained with slit-scanning topography and ultrasonic pachymetry [88].

Yusufoğlu et al. [87] showed an average percentage increase in CCT of about 2% in patients with DM compared to healthy controls and a reduction in the central corneal epithelial thickness (CCET) in diabetic patients with DR compared to those without DR (Table 2). In particular, CCET may decrease due to dry eye [89], impaired epithelial homeostasis associated with corneal neuropathy, and/or the effect of retinal photocoagulation [90].

Central corneal thickness may not correlate with the duration of DM and the presence of DR as suggested by the evidence of increased CCT regardless of the DR stage in two recent cross-sectional studies (Table 2) [86,88]. Interestingly, an analysis with AS-OCT of 100 diabetic eyes showed that the mean anterior chamber width (ACW) was narrower in eyes with NPDR than those with no DR [86], suggesting that ACW may be an adjunctive marker of DR. The increased oxidative stress and/or the reduced antioxidant capacities of diabetic eyes may lead to lens thickening, reduction in the anterior chamber volume and narrowing of the angle [91,92]. Based on ACW findings, in the absence of DR, diabetes itself might not be considered as a risk factor for primary angle closure glaucoma

#### 5.1.2. Ultrasound Pachymetry

Cross-sectional studies, in which ultrasound pachymetry was used to assess CCT, confirmed the significant increase in CCT in diabetic eyes compared to controls regardless of the DR severity (Table 2) [93,94]. CCT may correlate positively with levels of serum glucose and HbA1c [95], and DM duration and increased CCT is controversial (Table 2) [91,92,94].

#### 5.1.3. Specular Microscopy

The studies analyzing CCT with specular microscopy provided conflicting results on the effect of the duration of DM, HbA1c levels and severity of DR on CCT in diabetic patients (Table 2) [79,83,92,93,94,95,96,97,98,99,100,101,102]. In particular, with regard to HbA1c, on the one hand, the 3-month timeframe reflected by HbA1c might be too short to correlate with long-standing corneal alterations [103]; on the other hand, CCT can increase during acute hyperglycemia, and Hb1Ac is not a good marker of short-term blood glucose fluctuation [104].

#### 5.1.4. Dynamic Sheimpflug Analyzer Corvis ST (CST) and Pentacam

The comparison of diabetic versus healthy eyes in terms of spatial corneal thickness distribution showed that the values of pachy slope (indicator of the corneal thickness change from the apex to the periphery), thinnest corneal thickness (TCT) and peripheral pachymetry were significantly greater in the former, with no correlation with HbA1c value or DM duration [105]. Conversely, no CCT increase was reported [105].

### 5.2. Epithelial Cell Density

A lower basal epithelial cell density has been described in diabetic eyes, but the overall epithelial cell density may not significantly differ compared to that in healthy eyes [106,107,108]. This findings in diabetic eyes may be due an increased turnover rate of basal epithelial cells and the subsequent increased maturation and differentiation of superficial cells, compensating for the reduced basal epithelial cells density [109].

### 5.3. Endothelial Cell Density (ECD)

#### 5.3.1. Specular Microscopy

The vast majority of studies that investigated ECD through specular microscopy found this parameter to be lower in diabetic patients than healthy controls, with a reported reduction rate ranging from 3 to 5.3% (Table 3) [83,98,100,101,110,111,112,113,114]. No significant difference has been documented in a minority of studies [102,115,116], which is potentially due to the different glycemic status and different severity of the patients included (Table 3). Indeed, poor glycemic control, high HbA1c and longer duration of DM in diabetic patients may influence negatively ECD and cells morphology [109]. However, the evidence on the impact of DM duration, Hb1Ac levels and severity of DR is still controversial [83,98,113]. Finally, Módis et al. suggested a role of DM type on ECD reduction, as they reported a lower ECD in type I DM patients but no significant difference between type II DM patients and healthy controls [111].

#### 5.3.2. In Vivo Corneal Confocal Microscopy (CCM)

Consistently with the proved equivalency of CCM and specular microscopy proved in ECD measurements [117,118], the significant reduction in ECD in diabetic eyes compared with healthy controls has been confirmed by most of the studies conducted using CCM (Table 3) [106,107,119].

### 5.4. Coefficient of Variation in Cell Size (CV)

Coefficient of variation in cell size has been more commonly found to be greater in patient with diabetes compared with healthy controls [83,98,100,110,114,116,120], with the exception of a few studies that did not find any significant difference [112,113]. As for ECD, there is no agreement on the potential correlation between CV and duration of DM, Hb1A1c levels, or DR severity [93,98,120]. In particular, no statistically significant correlation between any of the above-mentioned parameters and CV was reported by El-Agamy et al. [98] In contrast, Lee et al. noted that CV was higher in patients with longer DM duration (more versus less than 10 years) [93]. Additionally, Taşlı et al. reported that CV correlated positively with HbA1c levels as well as the presence and progression of DR [120].

### 5.5. Percentage of Hexagonal Cells

The reduction in Hex is an established DM-related alteration [83,110,116,120], even if the difference with healthy eye has not always been found to be significant [98,100,113]. This may correlate negatively with DR presence and stage [110,120].

### 5.6. Diabetic Corneal Neuropathy

Diabetic corneal neuropathy is the ocular manifestation of the diabetic peripheral neuropathy (DPN), a long-term complication of DM, consisting of a length-dependent axonopathy that affects approximately 50% of diabetic patients [121]. So far, the diagnosis of DPN is clinical [122], and most of the methods currently used to define its severity only evaluate large nerve fiber function [123]. However, modifications in small nerve fibers are potentially a more sensitive marker of DPN, as these are involved earlier in the course of the disease [124,125]. In addition, the current gold standard to detect small nerve fibers, skin biopsy, is an invasive and non-repeatable procedure, which is not applicable to routine clinical practice [126]. Conversely, the analysis of corneal nerves thought in vivo CCM, a real-time and non-invasive imaging method, has the potential to offer a crucial opportunity to identify early nerves damage in diabetic patients [127,128]. Indeed, the cornea is the most densely innervated structure in the human body, and DM-induced metabolic stress leads to damage in corneal small nerve fibers in the early stages of DM [129].

#### In Vivo Corneal Confocal Microscopy

The parameters most frequently evaluated as possible biomarkers of DPN are corneal nerve fiber density (CNFD, the total number of main nerve fibers in a CCM image, expressed in fibers/mm^2^); corneal nerve branch density (CNBD, the number of branches connected to main nerve fibers, expressed in branches/mm^2^); corneal nerve fiber length (CNFL, the total length of all nerve fibers and branches per image, expressed in mm/mm^2^), and tortuosity of the main nerve fibers [130]. In particular, CNFD, CNFL and CNBD were reported to be significantly reduced in both type 1 and type 2 DM [107,131,132], and CNFL may be the parameter most strongly related to the severity of small nerve fiber neuropathy [133,134].

Diabetic corneal neuropathy may correlate with DPN [131,135,136], as supported by the demonstration of significant changes in CNFD, CNFL and CNBD in diabetic patients with the worsening of DPN [137,138]. In addition, recently, the rapid corneal fiber loss (RCNFL) defined by values exceeding the 5th percentile of 6% corneal fiber loss, has been proposed as new marker of DPN development as associated with progression of the impairment of large nerve fiber, even in absence of HbA1c changes [139]. Importantly, the demonstration of the same trend of progressive reduction in nerve fiber density, nerve branch density and nerve fiber length at intraepidermidal and corneal level with increasing severity of DPN supported the comparable diagnostic value of CCM and skin biopsy [140,141,142].

With regard to the potential correlation between DR and corneal nerve fiber changes, the evidence is still controversial. Corneal sub-basal nerve plexus alterations may be more severe in eyes with DR than in those without DR as well as correlate with DR stage [131]. In addition, corneal and intraepidermal nerve fiber loss was found to be more pronounced in advanced stages of DR [143]. Argon laser photocoagulation may contribute to the worsening of the corneal nerve damage [90]. The neuronal damage of both cornea and retina may occur in early stages of DR [144,145,146], specifically in terms of CNFL [144]. This underlines the importance of the examination of the corneal sub-basal nerve plexus when clinical signs of DR are absent. Furthermore, the higher concentration of antigen-presenting cells, including Langerhans cells and dendritic cells, in the cornea of diabetic eyes compared to healthy controls, negatively correlated with corneal nerve fiber density, may support a role of the inflammation in the development of diabetic corneal neuropathy [147].

Alterations in corneal nerve fiber may also correlate with complications of DM other than DR. For instance, a history of clinically known nephropathy has been found to be significantly associated with reduced CNFD, CNFL and CNB [130,148].

Finally, the significant improvement of CNFL 1 year after simultaneous pancreas and kidney transplantation in patients with DM type 1 may suggest that in vivo CCM may represent a precious tool also for the monitoring of diabetic therapy effectiveness [149,150].

## 6. Retinal Biomarkers

DR has been traditionally classified on the basis of specific retinal findings detectable on fundus examination, color fundus photography and fluorescein angiography, such as microaneurysms, dot and blot hemorrhages, venous beading, intraretinal microvascular anomalies (IRMA), vitreous/preretinal hemorrhage and neovascular [151].

### 6.1. Diabetic Macular Oedema

#### 6.1.1. OCT

The OCT plays an invaluable role in the assessment and management of DME, which is the main cause of moderate vision loss in patients affected by DR [152]. Multiple retinal OCT-biomarkers have been identified so far:

- Intraretinal cystoid spaces: The persistence of intraretinal cystoid spaces can result in permanent photoreceptor damage and visual impairment. Some findings of intraretinal cysts, including the location, size and presence of bridging hyperreflective material, have been associated with functional prognosis in diabetic eyes [153]. In particular, intraretinal cysts larger than 200 μm in the outer nuclear layer (ONL) have been associated with the disruption of IS/OS junction, reduced retinal sensitivity on microperimetry, poor visual prognosis and greater extent of macular ischemia [154,155,156]. The size of the cysts may also have a predictive value in case of pars plana vitrectomy (PPV) and internal limiting membrane (LM) peeling for chronic DME, as the presence of intraretinal cysts larger than 390 μm has been associated with the postoperative development of subfoveal atrophy [157].
- Increased retinal thickness: Although increased retinal thickness (RT) is strictly associated with the presence of subretinal and/or intraretinal fluid, these findings appeared to be not correlated with visual acuity and visual outcomes in eyes with DME [154].
- Hyperreflective retinal foci (HRF) is defined as intraretinal dots located in both inner and outer retina, with reflectivity similar to that of retinal nerve fiber layer, diameter <30 μm and no back-shadowing. These lesions may represent extravasated lipoproteins [158] or activated microglial cells [159] and are widely considered biomarkers of retinal inflammation [160]. It has been suggested as a better response to intravitreal dexamethasone implant compared to anti-VEGFs, but there is also a higher rate of recurrence in eyes with a higher number of HRF [161,162].
- Hard exudates: Differently from HRF, hard exudates are characterized by size >30 μm, back-shadowing, reflectivity similar to the RPE–Bruch’s membrane complex and location within the outer retinal layers. Conversely, hyperreflective dots with the same characteristics but located in the inner retina have been described as microaneurysms [163]. It has been suggested that hard exudates may be used as markers for treatment response in DME [164] and may be associated with better response to dexamethasone implant compared to intravitreal anti-VEGF agents [165].
- Disorganization of retinal inner layers: The presence of disorganization of retinal inner layers (DRIL) is evaluated in an area of 1 mm diameter centered on the foveal center. This finding has been associated with retinal dysfunction, even in case or early neuroretinal impairment [166]. An extent of DRIL of more than 50% of this area has been proposed as a negative prognostic factor for visual outcomes in eyes with DME before and/or after treatment [167]. In addition, DRIL may be associated with the presence of diabetic maculopathy regardless of the presence of DME, being correlated with the size of FAZ, the area of capillary non-perfusion, increased foveal thickness, the presence of EZ/ELM disruption and the severity of DR [168,169,170]. A negative correlation between RNFL thickness and DRIL has also been reported [171].
- Hyperreflective bridging retinal processes: It has been suggested that these processes between the cystic cavities represent neuronal tissue bridging between outer and inner retina [153]. Bridging retinal processes may be associated with better visual outcomes after anti-VEGF injections in eyes with DME [172], whereas eyes with no bridging retinal processes may be more likely to develop foveal atrophy post-treatment [173].
- Subfoveal neurosensory detachment (SND): The potential influence of SND on visual outcomes after intravitreal anti-VEGF agents for DME remains controversial [174,175,176,177]. This finding has been described in up to 30% of eyes with DME and may be correlated with the disruption of the external limiting membrane (ELM) that allow fluid and protein to migrate from the retina to the subretinal space [178]. In addition, the presence of SND may correlate with a greater amount of HF and a reduced retinal sensitivity [178]. Based on the detection of higher levels of IL-6 in eyes with SND, the latter has been proposed as a sign of retinal inflammation, and good response following dexamethasone implant has been reported [179,180]. A better response to aflibercept injection has also been reported in eyes with SND compared with those without SND [181].
- Alteration in outer retinal layers: The length of the photoreceptor outer segment may be reduced in patients with DR with or without DME compared to healthy eyes and may be a good indicator of visual acuity in eyes with DME [182,183]. As in other macular pathologies, the presence of preserved outer retinal layers, in particular an external limiting membrane (ELM) and ellipsoid (EZ) band appears to be associated with better visual outcomes in eyes with DME [179].

#### 6.1.2. Fluorescein Angiography (FA)

Fluorescein angiography has been traditionally used for the staging and management planning in DR and represents still a crucial diagnostic technique for the detection of leakage [184]. Diabetic maculopathy has been traditionally divided in focal, diffuse and ischemic based on the fluorangiographic appearance of the macula [185]. The former is characterized by focal leakage from microaneurysms surrounded by hard exudates; diffuse maculopathy manifests as a diffuse leakage involving the posterior pole in the early phase of FA. Finally, the latter refers to the presence of macular ischemia, which is characterized by an increase in the foveal avascular zone (FAZ) [186]. It is worth noting that FAZ is known to be enlarged and irregular in eyes with DR due to the occlusion of perifoveal capillaries regardless of the presence of ischemic maculopathy [187].

#### 6.1.3. OCT-Angiography (OCTA)

OCTA may be more sensitive to the detection of capillary non-perfusion areas compared to FA thanks to the absence of areas obscured by fluorescein leakage [188]. An enlargement of FAZ and, more specifically of the deep capillary plexus (DCP), has been reported in diabetic eyes, regardless of the presence of DR [189]. In addition, both superficial capillary plexus (SCP) and DCP appeared to be significantly reduced on fractal analysis and in terms of vessel density (VD) in patients with DM compared to healthy controls [190,191]. However, the reduction in VD of SCP and DCP may be more marked in eyes with DR compared to those without DR [192,193] as well as in eyes with DME compared to those without DME [194] and correlate with the degree of DR [192,193]. The reduction in VD in the DCP may also be associated with worse visual acuity and the progression of NPDR [195]. The analysis of the changes in both SCP and DCP has been performed before and after macular surgery in diabetic eyes, confirming that the DCP may be more sensible to vascular and iatrogenic damage [196,197]. In addition of the reduced VD, the non-perfusion area (NPA) may increase as the severity of DR increases [198]. Finally, an enlargement of FAZ may be associated with the presence of DRIL [199].

Recently, the perfused capillary density (PCD) has been suggested as a biomarker for DR as reported to be reduced in eyes with DR compared with those without DR, and this reduction may be more marked in eyes with PDR compared to eyes with NPDR [200].

### 6.2. Peripheral Retinal Ischemia

Ultra-widefield fluorescein angiography (UWFA) represents the standard imaging method used to evaluate the vascular changes in retinal periphery in diabetic eyes. The wider filed detectable with UWF photography and FA can be particularly useful in the assessment of DR, as peripheral retinal findings may correlate with a higher risk of DR progression [184,201].

Retinal ischemic areas, characterized by the absence of visible retinal vasculature, can be delimited by tortuous, dilated, shunt vessels. The extent of peripheral retinal ischemia may correlate with DME [202,203]. The ischemia index has been defined as the percentage of the extent of ischemic areas of ischemia out of the total retinal area and has been suggested as biomarker of DR activity [204,205,206].

Recently, the retinal vascular bed area (RVBA), measured as the automatic sum of the real size (in mm^2^) of all the pixels, has been proposed as a new biomarker for the efficiency of retinal vascular changes following anti-VEGF injection [207]. In particular, a reduction on RVBA may be present in eyes with PDR and significant ischemia after anti-VEGF treatment [207].

### 6.3. IRMA and Neovessels

The differential diagnosis between IRMA and retinal neovascularization is crucial to distinguish between NPDR and PDR and, thus, for the patient management and prognosis.

FA represents the mainstay to detect neovessels and, in particular, for the differential diagnosis between neovessels and IRMA. Indeed, the former are characterized by an early and intense focal leakage, whereas the latter do not show leakage on FA. However, recent studies supported the role of OCTA for the detection of neovessels and their changes following treatment [208].

## 7. Conclusions

Over the last decades, the developments in ocular imaging allowed us to assess the structure of several ocular tissue at a near-histological level at high resolution and in a non-invasive manner. The management of DR has significantly benefited from the identification of objective, quantifiable signs, used as biomarkers, of greater relevance in clinical practice and research for diagnosis, prognosis and treatment planning. In addition, the association between some of these biomarkers with early or even subclinical stages of the disease, has the potential to result in a significant contribution in the screening protocols and, thus, prevention of the development and progression of DR and the optimization of visual outcomes. Along with the ocular imaging biomarkers, biomarkers detectable in the serum/plasma and ocular fluid are playing a crucial role not only for the diagnosis and prognosis of DM and DR but also for the insights provided in the understanding of DR pathogenesis.

Despite the promising applications in clinical practice and research, the acquisition technique as well as the operator-dependent evaluation can still represent a limitation for some imaging methodologies; in this regard, the development of automated analysis and deep learning algorithms capable of a fast, reproducible and accurate image segmentation can overcome these limitations.

## Figures and Tables

**Table 1:** Angiogenic and inflammatory mediators in diabetic retinopathy.

Molecules	Vitreous	Aqueous
Upregulated pro-angiogenic mediators	VEGF, FGF-2, Ang-1, Ang-2, PDGF, EPO, osteopontin, PlGF, CTGF, IGF, CYR61, SDF-1, HGF, LRG1	VEGF, PlGF, PDGF, HGF
Downregulated anti-angiogenic mediators	PEDF, TSP-1	PEDF
Upregulated pro-inflammatory mediators	TNF-α, IL-1α, IL-8, IL-6, IP-10, MCP-1, CRP, IL-12, IL-15, IL-16, IL-18	MCP-1, IL-1b, IL-6, IL-8, IL-12, IP-10
Other molecules—upregulated		glutamine, histidine, threonine, asparagine, PTX3
Other molecules—downregulated	NGF, BDNF, NT-3, NT-4, CNTF, GDNF	lactate, succinate, IL-10

Ang-1, angiopoietin-1; Ang-2, angiopoietin-2; BDNF, brain-derived neurotrophic factor; CNTF, ciliary neurotrophic factor; CRP, c-reactive protein; CTGF, connective tissue growth factor; CYR61, cysteine-rich 61; EPO, erythropoietin; FGF-2, fibroblast growth factor 2; GDNF, glial cell-derived neurotrophic factor; HGF, hepatocyte growth factor; ICAM-1, intracellular adhesion molecule type 1; IGF, insulin-like growth factor; IL, interleukin; IP-10, interferon-α-inducible protein-10; LRG1, leucine-rich α-2-glycoprotein; MCP-1, monocyte chemoattractant protein-1; NGF, nerve growth factor; NT, neurotrophin; PDGF, platelet-derived growth factor; PEDF, pigment epithelium-derived factor; PlGF, placental growth factor; SDF-1, stromal cell-derived factor 1; TNF-α, tumor necrosis factor-α; TSP-1, thrombospondin 1; VEGF, vascular endothelial growth factor.

**Table 2:** Principal studies comparing central corneal thickness (CCT) in diabetic and non-diabetic patients.

Authors, Years	Study Design	Imaging Method	Eyes (*n*)		CCT (µm)		Conclusions
			DM	Controls	DM	Controls	
Suraida et al., 2018 [86]	CS	AS-OCT	DM = 100 NoDR = 50 NPDR = 50	50	524.60 ± 28.74 529.26 ± 33.88	493.12 ± 67.08	Diabetic patients appear to have significantly thicker CCT regardless the retinopathy status (*p* < 0.001)
Yusufoglu et al., 2022 [87]	P, CS	AS-OCT	72	72	544.33 ± 31.20	533.77 ± 24.45	The CCT was statistically significantly thicker in diabetic patients than in the controls (*p* = 0.025)
Canan et al., 2020 [88]	P, CS	AS-OCT SST UP	NoDR = 49 NPDR = 30 PDR = 17 NoDR = 49 NPDR = 30 PDR = 17 NoDR = 49 NPDR = 30 PDR = 17		521.71 ± 27.58 528.20 ± 29.16 516.94 ± 34.25 568.10 ± 32.5 567.57 ± 35.49 554.47 ± 25.95 551.1 ± 29.64 556.07 ± 31.18 544.18 ± 36.33		No correlation between CCT and the severity of retinopathy (*p* > 0.05) Better correlation for OCT and UP.
Lee et al., 2006 [93]	CS	UP	200 ≤10y = 111 >10y = 89	100	588.2 ± 2.7 582.2 ± 3.7 595.9 ± 4.2	567.8 ± 3.8	Diabetic patients show significantly higher CCT differences compared to controls (*p* < 0.05) DM of over 10 years’ duration showed thicker corneas (*p* < 0.05)
Özdamar et al., 2010 [92]	CS	UP	DM = 100 NoDR = 29 NPDR = 48 PDR = 23	145	564 ± 30 565 ± 32 558 ± 31 582 ± 23	538 ± 35	The CCT of diabetic patients is thicker when compared with non-diabetic patients (*p* = 0.001) Differences between DM subgroups are not statistically significant (*p* = 0.056)
Su et al., 2008 [94]	CS	UP	748	2491	547.2 ± 1.2	539.3 ± 0.7	Thicker corneas in patients with DM (*p* < 0.001)
Galgauskas et al., 2016 [97]	P, CS	NCSM	123	120	566.7 ± 35.7	550.0 ± 56.4	CCT is significantly higher in diabetic patients (*p* < 0.05)
El-Agamy et al., 2020 [98]	P, CS	NCSM	DM 2 = 57	45	545.61 ± 30.39	539.42 ± 29.22	No significant difference in CCT between diabetic and control groups (*p* = 0.301)
Inoue et al., 2002 [100]	CS	UP	DM 2 = 99	97	538 ± 36	537 ± 38	CCT is not increased in type II DM (*p* = 0.90)
Urban et al., 2013 [101]	CS	NCSM	DM 1 = 123	124	550 ± 30	530 ± 33	CCT is increased in children and adolescents with DM (*p* < 0.0001)
Storr-Paulsen et al., 2014 [102]	P, CS	NCSM	107	128	546 ± 7	538 ± 5	Diabetic patients show a significant increase in CCT (*p* < 0.05)
Ramm et al., 2020 [105]	P, CS	Pentacam Corvis ST	59	57	552.6 ± 33.2 553.4 ± 35	552 ± 36.6 558 ± 38.6	No significant increase in CCT in diabetic patients (*p* = 0.923 and *p* = 0.511 with Pentacam and Corvis, respectively)

AS-OCT: anterior segment optical coherence tomography; CS: cross-sectional; DM: diabetes mellitus; DR: diabetic retinopathy; NCSM, noncontact specular microscope; NPDR: non-proliferative diabetic retinopathy; P: prospective; PDR: proliferative diabetic retinopathy; SM: specular microscope; SST: slit-scanning topographer; UP: ultrasonic pachymeter.

**Table 3:** Studies comparing endothelial cell density in diabetic and non-diabetic patients.

Authors, Years	Study Design	Imaging Method	Eyes (*n*)		ECD Cell/mm²		Conclusions
			DM	Controls	DM	Controls	
Choo et al., 2010 [83]	CS	NCSM	DM 2 = 100	100	2541.6 ± 516.4	2660.1 ± 515.5	ECD in DM2 group was significantly lower than in the control group (*p* < 0.05)
El-Agamy et al., 2020 [98]	CS	NCSM	DM 2 = 57	45	2491.98 ± 261.08	2629.68 ± 293.45	ECD was significantly lower in the diabetic cornea than in the control group (*p* = 0.014)
Inoue et al., 2002 [100]	CS	SM	DM 2 = 99	97	2493 ± 330	2599 ± 278	ECD was significantly lower in the diabetic cornea than in the control group (*p* = 0.016)
Urban et al., 2013 [101]	CS	NCSM	DM 1 = 123	124	2435.55 ± 443.43	2970.75 ± 270.1	ECD was significantly lower in patients with diabetes than in the control group (*p* = 0.0001)
Jha et al., 2022 [110]	CS	NCSM	DM 2 = 592	596	2484.5 ± 299.5	2555.9 ± 258.2	ECD was significantly lower in the diabetic cornea than in the control group (*p* = 0.017)
Modis et al., 2010 [111]	CS	NCSM	DM 1 = 41 DM 2 = 59	N/A	2428 ± 219 2495 ± 191	N/A	ECD was significantly lower in the diabetic cornea than in the control group (*p* = 0.02). No significant differences between DM2 and controls
Sudhir et al., 2012 [112]	CS, P	NCSM	1191	120	2550.96 ± 326.17	2634.44 ± 256.0	ECD was significantly lower in the diabetic cornea than in the control group (*p* = 0.001).
Islam et al., 2017 [113]	CS	NCSM	149	149	2494.47 ± 394.10	2574.46 ± 279.97	ECD was significantly lower in the diabetic cornea than in the control group (*p* = 0.04).
Storr-Paulsen et al., 2014 [102]	CS, P	NCSM	DM 2 = 107	128	2578 ± 77	2605 ± 66	No differences in ECD between well-controlled diabetic subjects and non-diabetic subjects (*p* = 0.60)
Quadrado et al., 2006 [106]	CS, P	CCM	15	15	2660 ± 364	2690 ± 302	ECD in diabetic patients is not significantly different from healthy controls (*p* = 0.5)
Szalai et al., 2016 [107]	CS	CCM	No DR = 10 DR = 18	17	3250.36 ± 421.5 2639.17 ± 227.5	3497.62 ± 519.8	ECD was significantly lower in patients with DM without and with retinopathy compared to control subjects (*p* = 0.001)
Shenoy et al., 2009 [119]	Cohort study	CCM	110	110	2342 ± 392	2517 ± 647	ECD was significantly lower in the diabetic cornea than in the control group

CCM: corneal confocal microscopy; CS: cross-sectional; DM: diabetes mellitus; DR: diabetic retinopathy; NCSM, noncontact specular microscope; NPDR: non-proliferative diabetic retinopathy; P: prospective; PDR: proliferative diabetic retinopathy; SM: specular microscope.

## Data Availability

Not applicable.
